# Multivariate Quantification of the Solid State Phase Composition of Co-Amorphous Naproxen-Indomethacin

**DOI:** 10.3390/molecules201019571

**Published:** 2015-10-27

**Authors:** Andreas Beyer, Holger Grohganz, Korbinian Löbmann, Thomas Rades, Claudia S. Leopold

**Affiliations:** 1 Division of Pharmaceutical Technology, University of Hamburg, Hamburg 20146, Germany; E-Mail: andreas.beyer@chemie.uni-hamburg.de; 2 Department of Pharmacy, University of Copenhagen, Copenhagen 2100, Denmark; E-Mails: holger.grohganz@sund.ku.dk (H.G.); korbinian.loebmann@sund.ku.dk (K.L.); thomas.rades@sund.ku.dk (T.R.)

**Keywords:** co-amorphous, solid state analysis, composition, multivariate, PLS, XRPD, recrystallization, stability

## Abstract

To benefit from the optimized dissolution properties of active pharmaceutical ingredients in their amorphous forms, co-amorphisation as a viable tool to stabilize these amorphous phases is of both academic and industrial interest. Reports dealing with the physical stability and recrystallization behavior of co-amorphous systems are however limited to qualitative evaluations based on the corresponding X-ray powder diffractograms. Therefore, the objective of the study was to develop a quantification model based on X-ray powder diffractometry (XRPD), followed by a multivariate partial least squares regression approach that enables the simultaneous determination of up to four solid state fractions: crystalline naproxen, γ-indomethacin, α-indomethacin as well as co-amorphous naproxen-indomethacin. For this purpose, a calibration set that covers the whole range of possible combinations of the four components was prepared and analyzed by XRPD. In order to test the model performances, leave-one-out cross validation was performed and revealed root mean square errors of validation between 3.11% and 3.45% for the crystalline molar fractions and 5.57% for the co-amorphous molar fraction. In summary, even four solid state phases, involving one co-amorphous phase, can be quantified with this XRPD data-based approach.

## 1. Introduction

Poorly water soluble active pharmaceutical ingredients (APIs) present an increasing challenge for the development of solid oral dosage forms, as the percentage of those substances entering the development phase has been estimated to be up to 60%–70% [1,2]. A sufficient aqueous solubility of a potential API is essential for its oral bioavailability, as poor solubility is likely to result in insufficient absorption [3]. One approach to increase the API solubility is the transformation of crystalline compounds into their amorphous form [4], which presents a promising method on the supramolecular level to improve the dissolution profile and hence the oral bioavailability of APIs [5,6].

However, the inherent physical instability of amorphous systems manifesting itself in recrystallization during manufacturing, processing, storage and administration is still the main disadvantage of this approach [7,8]. To overcome the present limitations in the application of amorphous solids, the respective API is frequently blended with amorphous pharmaceutical polymers, resulting in solid polymer dispersions, in which the compound is homogenously and molecularly dispersed within a hydrophilic polymer matrix (glass solution) [9,10]. A key parameter describing the improved physical stability of solid polymer dispersions is the increased glass transition temperature of the mixture compared to the plain amorphous drug [5,11]. However, the main disadvantages of solid polymer dispersions are, firstly, the hygroscopicity of the involved polymers, which may lead to water absorption and thereby decrease the physical stability of the systems [12], and secondly, limitations concerning the miscibility of APIs and polymers, which may result in unfavorable API-polymer ratios [5]. Furthermore, challenges during pulverization, processing into dosage forms and scale up of solid polymer dispersions are reported [5,13,14,15].

Thus, there is a need to develop an alternative approach to amorphous API-polymer systems, which led to the concept of co-amorphous systems [16]. These systems are the result of the simultaneous conversion of at least two crystalline small molecule compounds into one amorphous phase that is homogeneous on a molecular level. Co-amorphous systems consisting of either two pharmacologically matching APIs [17,18], two excipients [19] or an API plus excipient [20,21] and combinations involving amino acids in binary and ternary mixtures [22,23,24] are already described in the literature. Co-amorphous systems show an improved physical stability and also have the potential to further improve the API solubility [16]. For most of these systems [25], the advantages regarding the improved physical stability were attributed to distinct intermolecular interactions between the blended compounds [16,26] that resulted in the formation of heterodimers [16]. It was shown that the ratio of the molecules present in the co-amorphous phase plays a more important role with regard to the physical stability of these systems than the glass transition temperature (T_g_), *i.e.*, in most of the studies, the best physical stability was found with the respective equimolar systems [16].

To date, stability studies regarding the recrystallization behavior of co-amorphous systems were only based on qualitative evaluations of the emerging peak shifts in the respective FTIR spectra [25] or peak intensities in the respective X-ray powder diffractograms [17,18,25,27]. However, X-ray powder diffractometry (XRPD) also offers the opportunity to analyze the phase composition of solids in a quantitative manner.

XRPD is able to detect periodic molecular structures [28] in a given material because of constructive X-ray beam diffraction that occurs if Bragg’s law is applicable [29]. Therefore, crystalline structures lead to distinct peak intensities while non-periodic samples such as amorphous solids show halo signals in the diffractograms [7]. The classical evaluation methods to quantify multicomponent samples based on XRPD data are the relative intensity ratio (RIR) method and the Rietveld method [30].

The RIR method takes the intensities of the XRPD signals of the involved crystalline and amorphous components into account as these are proportional to the fractions of the respective phases [31]. The RIR method is easy to use but may be limited regarding its accuracy [32,33] or if the peaks present in the diffractograms are not well separated [34].

Compared to the RIR method, the Rietveld method [35] presents the more accurate approach [33], which is based on a crystal structure model that is varied until a maximal fit of the hereby calculated theoretical and the recorded diffractogram is achieved [30]. Therefore, the quantification of an amorphous fraction using the Rietveld method is only indirectly possible [33] and it generally requires knowledge of the crystal structures of the crystalline components [36].

As an alternative method that provides determination accuracies comparable to those of the Rietveld method [37] but does not require knowledge of the crystal structure, multivariate partial least squares (PLS) regression may be used to quantify the multiphase composition of a given sample.

Rumondor *et al.* [32] showed that this approach was successful to quantify the crystalline felodipine fraction in blends of felodipine and its solid glass solution with polyvinylpyrrolidone and led to accurate predictions with significantly lower root mean square errors compared to results obtained by application of the RIR method. Furthermore, Caliandro *et al.* [37] reported that the combination of XRPD and PLS can deliver accurate results even for the quantification of mixtures that comprised four crystalline phases at the same time with accuracies comparable to those of the Rietveld method.

To our knowledge no quantification of the phase composition of a quaternary multiphase system involving a co-amorphous phase has been carried out. Therefore, in the present study, co-amorphous naproxen-indomethacin [27] was chosen as a model system to develop several multivariate PLS regression models based on XRPD.

## 2. Results and Discussion

### 2.1. Determination of the Phase Composition of Co-Amorphous Naproxen-Indomethacin

**Figure 1:**
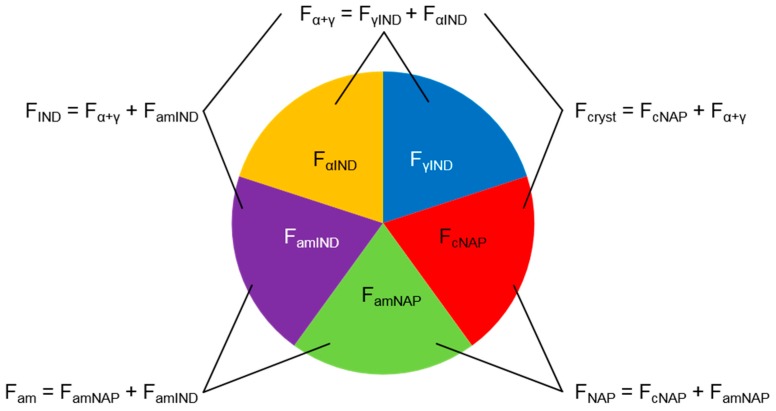
Circular chart showing the phases that can be present during the recrystallization of co-amorphous naproxen-indomethacin. The different colors represent the molar fractions of cNAP (F_cNAP_), γ-IND (F_γIND_), α-IND (F_αIND_), amorphous NAP (F_amNAP_) and amorphous IND (F_amIND_). F_am_ represents the total molar amorphous fraction, F_α+γ_ the total molar crystalline IND fraction, F_cryst_ the total molar crystalline fraction, F_IND_ the total molar IND fraction and F_NAP_ the total molar NAP fraction.

During the recrystallization of co-amorphous naproxen-indomethacin (aNAP/IND), the formation of crystalline naproxen (cNAP), γ-indomethacin (γ-IND) and α-indomethacin (α-IND) may occur, *i.e.*, up to four solid state phases can be present at the same time. Thus, in the respective diffractograms, the intensity of the halo signal resulting from the co-amorphous portion decreases over time, while the peak intensities resulting from the crystalline components increase. To fully characterize the solid-state phase composition of aNAP/IND samples, the determination of the molar fractions (F) that are present as cNAP, γ-IND and α-IND is sufficient. Taking the total naproxen (F_NAP_) and indomethacin fraction (F_IND_) into account, that are known by weight, the amorphous NAP fraction (F_amNAP_) and the amorphous IND fraction (F_amIND_) that together form the total (co-)amorphous fraction F_am_ can be determined according to the equations in Figure 1. Furthermore, the total molar crystalline IND fraction F_α+γ_ and the total molar crystalline fraction F_cryst_ may be predicted (Figure 1).

### 2.2. Molar Crystalline Naproxen Fraction F_cNAP_

**Figure 2:**
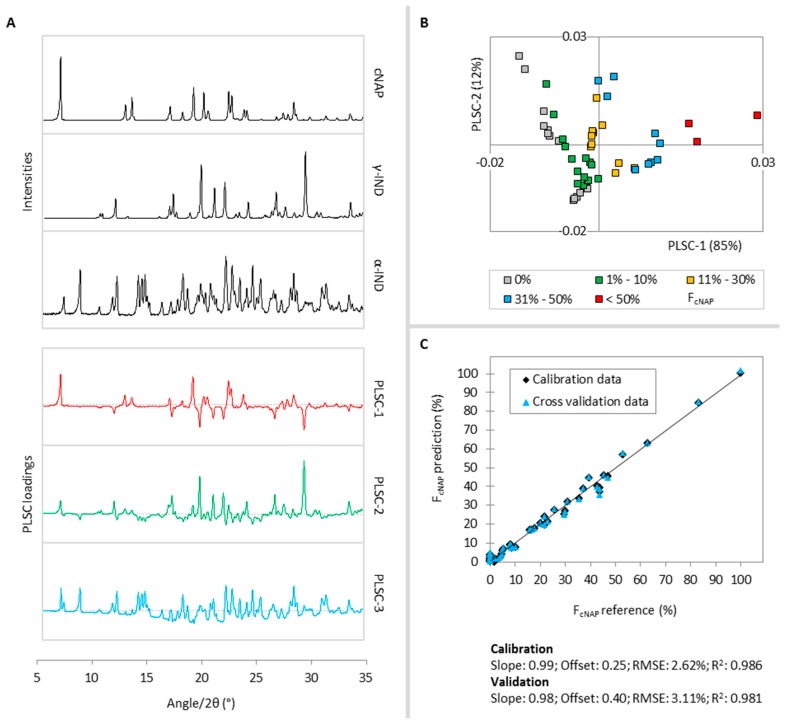
(**A**) Reference diffractograms of cNAP, γ-IND, α-IND (black lines) and PLSC-1 (red), PLSC-2 (green) and PLSC-3 (blue) loadings plots for the F_cNAP_ PLS model,; (**B**) PLSC-1-*vs.*-PLSC-2 scores plot, the different colors classify the calibration samples according to their cNAP fractions; (**C**) Correlation of the PLS predicted cNAP fractions *vs.* the reference values during the calibration (black diamonds) and cross validation (blue triangles).

Three PLS components (PLSCs) were found to describe 99% of the cNAP variance in the calibration samples. In Figure 2A, the reference diffractograms of cNAP, γ-IND, α-IND and the first three PLSC loadings plots are shown. PLSC-1 (85%) distinguishes cNAP diffraction signals (positive part) from γ-IND and α-IND intensities (negative part), while PLSC-2 (12%) distinguishes cNAP and γ-IND diffraction signals (positive part) from α-IND and halo intensities (negative part). Thus, the positive parts of both PLSCs contribute significant information to describe the cNAP fraction of the samples and therefore samples with high cNAP fractions cluster in the positive part in the PLSC-1-*vs.*-PLSC-2 scores plot, while samples with low or no cNAP fraction locate in quadrant two and three (Figure 2B). PLSC-3 (2%) separates α-IND and some small cNAP signals (positive part) from halo intensities (negative part) and thus only contributes little information to describe the cNAP fraction in the samples.

Comparison of the PLS predicted molar fractions of cNAP *vs.* the reference values reveals linearity between 0% and 100%, a goodness of fit (R^2^) of 0.986 and a root mean square error (RMSE) of 2.62%. These values change only slightly to 0.981% and 3.11%, respectively, during cross validation and confirm good model performance (Figure 2C).

### 2.3. Molar γ-Indomethacin Fraction F_γIND_

**Figure 3:**
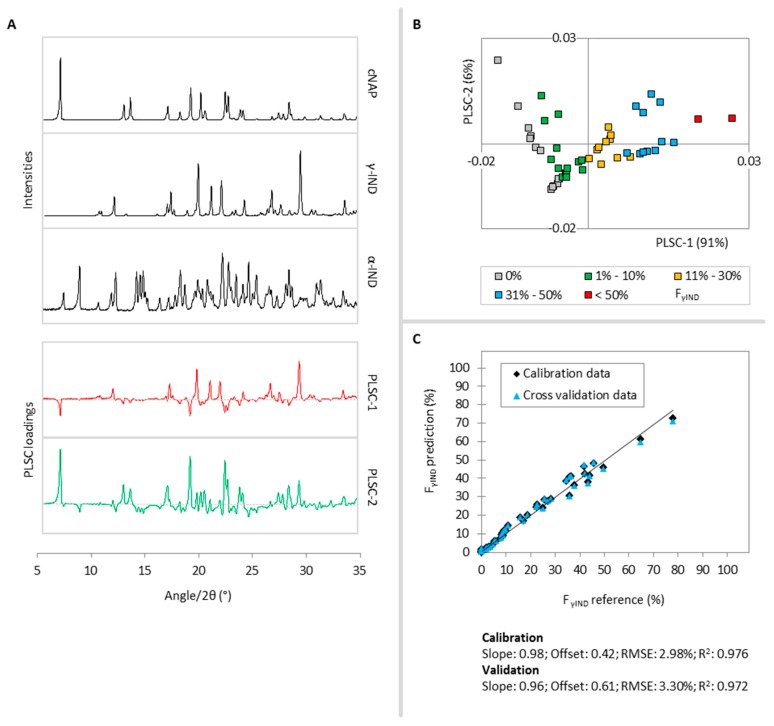
(**A**) Reference diffractograms of cNAP, γ-IND, α-IND (black lines) and PLSC-1 (red) and PLSC-2 (green) loadings plots for the F_γIND_ PLS model; (**B**) PLSC-1-*vs.*-PLSC-2 scores plot, the different colors classify the calibration samples according to their γ-IND fractions; (**C**) Correlation of the PLS predicted γ-IND fraction *vs.* the reference values during the calibration (black diamonds) and cross validation (blue triangles).

Two PLSCs were found to describe 98% of the γ-IND variance in the calibration samples. According to Figure 3A, PLSC-1 (91%) distinguishes γ-IND diffraction signals (positive part) from cNAP and some α-IND intensities (negative part) and PLSC-2 (6%) distinguishes γ-IND and cNAP diffraction signals (positive part) from α-IND and halo intensities (negative part). Thus, the positive parts of both PLSCs contain information to describe the γ-IND fraction of the samples and therefore, as it is also the case for the cNAP PLS model, samples with higher γ-IND content cluster in the first and fourth quadrant of the PLSC-1-*vs.*-PLSC-2 scores plot, while samples with small or no γ-IND fraction cluster in the second and third quadrant (Figure 3B).

In Figure 3C, the predicted values of F_γIND_ are plotted *vs.* the reference molar fractions. A linear correlation was found for γ-IND fractions between 0% and 78.1% and therefore, calibration sample 49 (Appendix A1: F_γIND_ = 100%) was excluded for the construction of the γ-IND PLS model. Thus, the γ-IND PLS model is not applicable for samples with γ-IND fractions higher than 78.1%. The R^2^ of 0.976 and the RMSE of 2.98% reveal a good model performance and only slightly change during the cross validation (R^2^: 0.972; RMSE: 3.30%).

### 2.4. Molar α-Indomethacin Fraction F_αIND_

**Figure 4:**
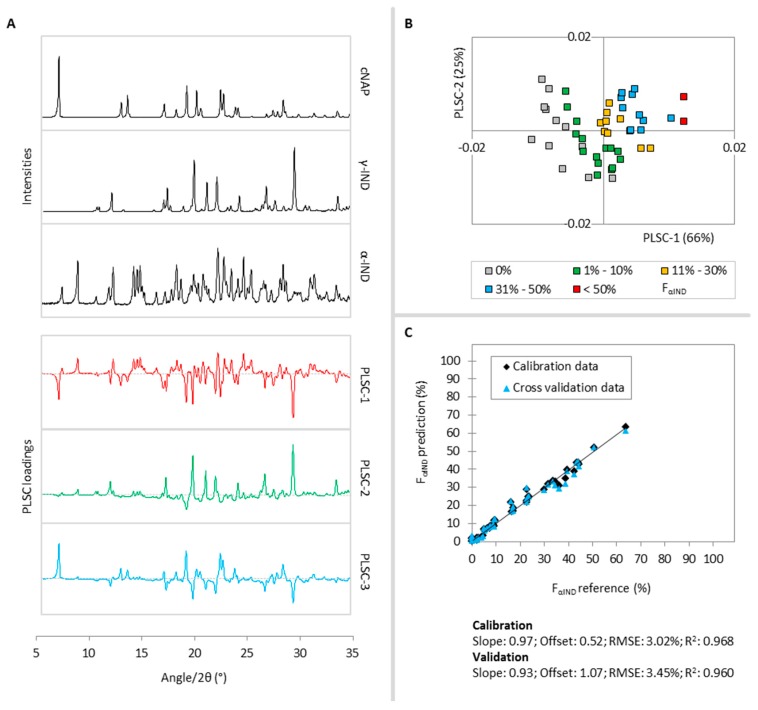
(**A**) Reference diffractograms of cNAP, γ-IND, α-IND (black lines) and PLSC-1 (red), PLSC-2 (green) and PLSC-3 (blue) loadings plots for the F_α-IND_ PLS model,; (**B**) PLSC-1-*vs.*-PLSC-2 scores plot, the different colors classify the calibration samples according to their α-IND fractions; (**C**) Correlation of the PLS predicted α-IND fraction *vs.* the reference values during the calibration (black diamonds) and cross validation (blue triangles).

Three PLS components were found to describe 97% of the α-IND variance in the calibration samples. According to Figure 4A, PLSC-1 (66%) distinguishes α-IND and halo diffraction signals (positive part) from γ-IND and cNAP intensities (negative part) and PLSC-2 (25%) distinguishes α-IND and γ-IND diffraction signals (positive part) from halo intensities (negative part). Both positive parts of the PLSC-1 and PLSC-2 contain significant information to describe the α-IND fraction of the samples and therefore, samples with higher α-IND content cluster in the first and fourth quadrant of the PLSC-1-*vs.*-PLSC-2 scores plot (Figure 4B). PLSC-3 (6%) separates some small α-IND and cNAP diffraction signals (positive part) from γ-IND and halo intensities (negative part) and thus further specifies the α-IND fraction of the samples.

In Figure 4C, the PLS predicted molar fractions of F_αIND_ are plotted *vs.* their respective reference values. A linear correlation was found for α-IND fractions between 0% and 63.8% and thus, calibration samples 22 and 48 (Appendix A1: F_αIND_ = 79.3% and 100.0%) were excluded for the construction of the α-IND PLS model. The α-IND PLS model is therefore not applicable for samples with α-IND fractions higher than 63.8%. The R^2^ of 0.968 and RMSE of 3.02% change only slightly to 0.960% and 3.45% respectively, during the cross validation, revealing a satisfactory model performance.

### 2.5. Total Molar Amorphous Fraction F_am_

With decreasing peak intensities of the crystalline components in the XRPD data, the shape of the processed diffractograms changes towards a halo, representing amorphous samples. Based on this fact, it was investigated if the total molar amorphous fraction F_am_ may be directly quantifiable by construction of a fourth PLS model.

**Figure 5:**
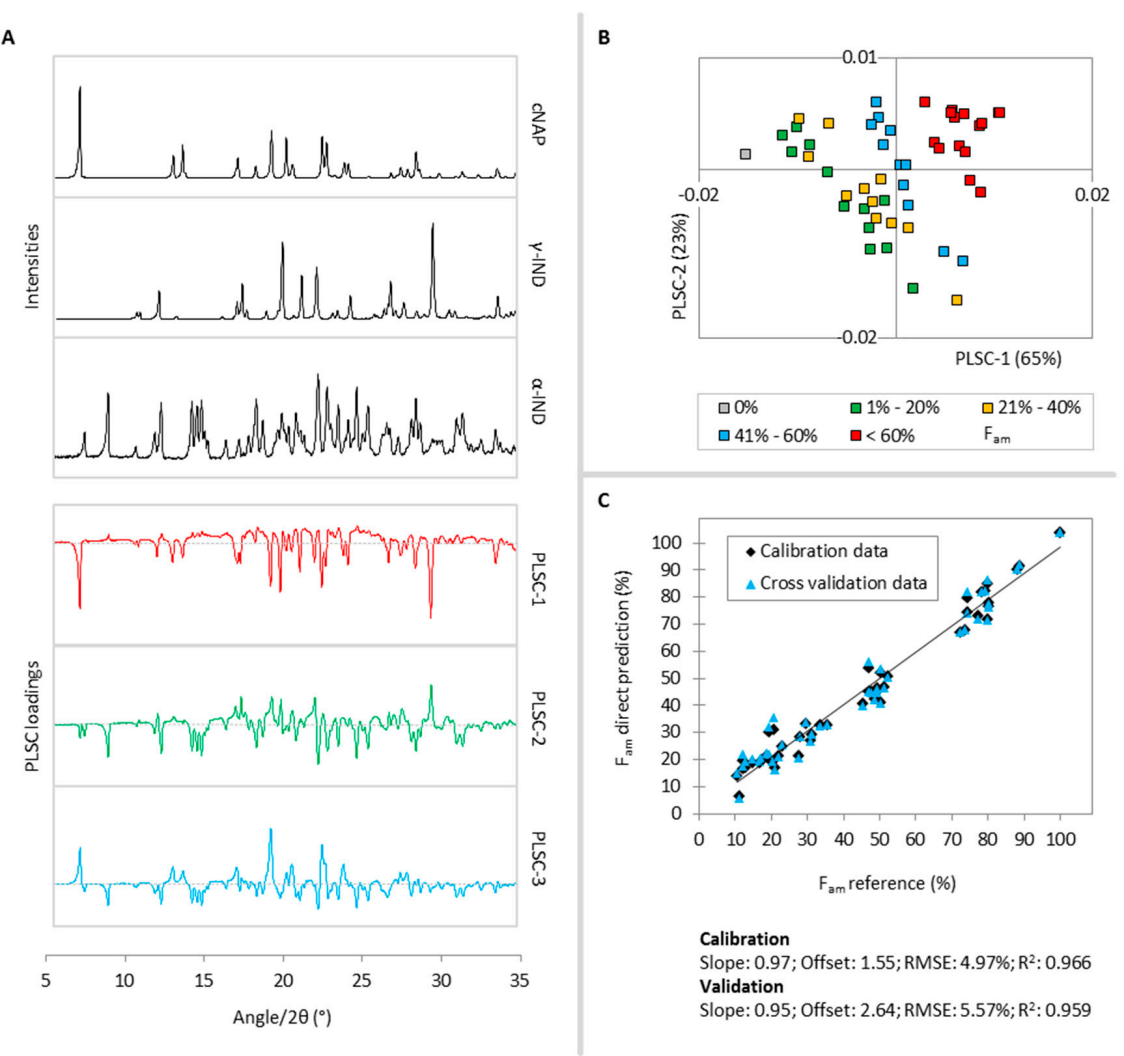
(**A**) Reference diffractograms of cNAP, γ-IND, α-IND (black lines) and PLSC-1 (red), PLSC-2 (green) and PLSC-3 (blue) loadings plots for the F_am_ PLS model; (**B**) PLSC-1-*vs.*-PLSC-2 scores plot, the different colors classify the calibration samples according to their amorphous fractions; (**C**) Correlation of the PLS predicted amorphous fraction *vs.* the reference values during the calibration (black diamonds) and cross validation (blue triangles).

Again, three PLSCs were found to describe 97% of the variability of the total molar amorphous fraction in the calibration set. In Figure 5A, the loadings plots of the first three PLSCs are shown. PLSC-1 (65%) distinguishes halo and α-IND diffraction signals (positive part) from γ-IND and cNAP intensities (negative part), while PLSC-2 (23%) separates γ-IND and halo diffraction signals (positive part) from α-IND and cNAP intensities (negative part). Therefore, samples with high amorphous fractions cluster in the positive parts of both PLSCs in the PLSC-1-*vs.*-PLSC-2 scores plot (Figure 5B). PLSC-3 (9%) distinguishes some halo and cNAP signals (positive part) from α-IND signals (negative part) and thus further specifies the co-amorphous fraction in the samples.

Comparison of the PLS predicted amorphous fractions *vs.* the true values (Figure 5C) reveals linearity between 10% and 100%. For fully crystalline calibration samples that contain exclusively cNAP, γ-IND or α-IND (Appendix A1: samples 48–50) the amorphous fractions were strongly overestimated to up to 20% and therefore, PLS predicted F_am_ values near or below 20% have to be considered carefully. The F_am_ PLS model is thus not applicable for fully crystalline samples. To exclude the presence of fully crystalline samples, the respective diffractograms should be checked for the presence of a halo signal. The R^2^ (0.966) and RMSE (4.97%) are slightly different compared to those of the other models. However, as XRPD as a measurement technique is best suited to describe the crystallinity of a sample, this is expected and the obtained model for the quantification of an amorphous phase can still be regarded as very good. The descriptors again change moderately during the cross validation (R^2^: 0.959; RMSE: 5.57%).

**Figure 6:**
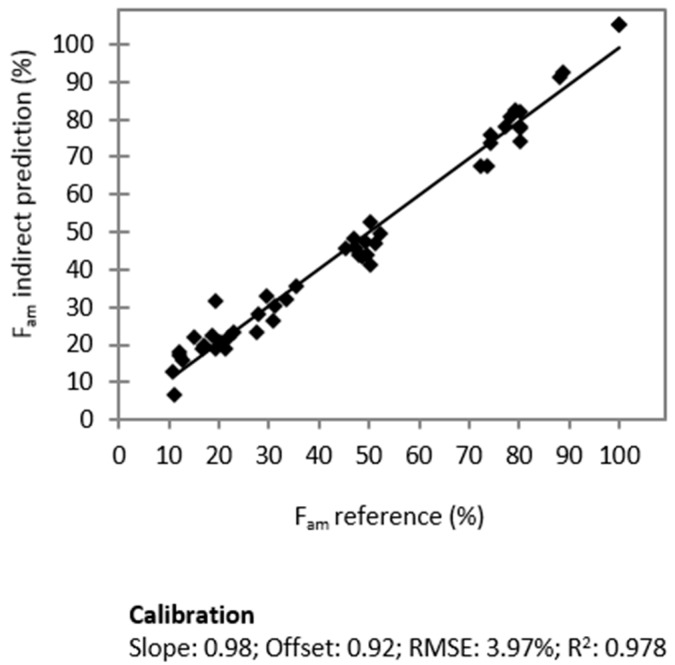
Correlation of the indirectly predicted amorphous fraction *vs.* the reference values.

The PLS model for the prediction of the amorphous contents can by verified by comparing the obtained values of this model with remains of the sum of the crystalline models (indirect prediction). For comparison, the indirectly predicted values for F_am_ according to Equation (1) are plotted *vs.* the reference fractions in Figure 6:

(1) $${\text{F}}_{\text{am}}=100-\left({\text{F}}_{\text{cNAP}}+{\text{F}}_{\text{γIND}}+{\text{F}}_{\text{αIND}}\right)$$

It can be seen that both methods deliver comparable results, although the indirect approach is slightly more accurate (R^2^: 0.978; RMSE: 3.97%), as could have been expected based on the principle of the measurement technique.

**Figure 7:**
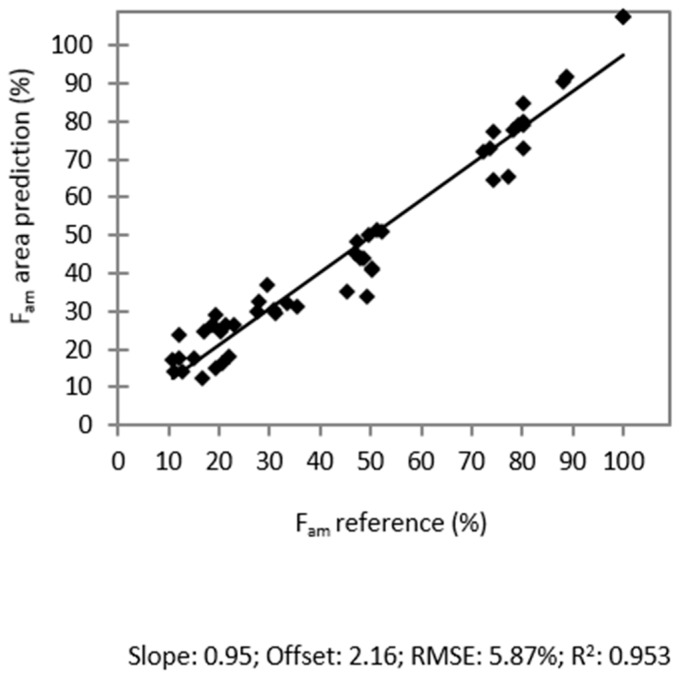
Correlation of the predicted amorphous fractions based on the relative area under the Bragg peaks *vs.* the reference values.

For comparison, F_am_ was also predicted based on the relative area (A) under the Bragg peaks in the diffractograms. Correlation of the true F_am_ values of the calibration samples *vs.* the predicted A values and linear regression resulted in a calibration function. The predicted values for F_am_ using the calibration function are plotted *vs.* the reference fractions in Figure 7.

R^2^ (0.953) and the RMSE (5.87%) were slightly worse compared to the results of the other two approaches.

## 3. Experimental Section 

### 3.1. Materials

Naproxen (NAP, M = 230.26 g/mol) and γ-indomethacin (M = 357.79 g/mol) were purchased from Fagron GmbH & Co. KG (Barsbüttel, Germany) and used as received. The α-IND polymorph was prepared by precipitation from an ethanolic solution by addition of water according to Atef *et al.* [38] before vacuum*-*drying (P_2_O_5_) and sieving (250 μm) was performed*.* Equimolar co-amorphous naproxen-indomethacin (aNAP/IND_1:1_) was prepared by quench-cooling the melt of the physical mixture of cNAP and γ-IND according to Löbmann *et al.* [27]. Subsequently, the resulting solid was crushed using a mortar and pestle before it was sieved (250 μm) in an air conditioned room at 6 °C. 

### 3.2. Methods

#### 3.2.1. Partial Least Squares Regression

Multivariate PLS regression was chosen to quantify each of the molar fractions in the quaternary blends based on XRPD data. This projection method is able to extract the most relevant information of a given dataset by reduction of its dimensionality. An XRPD file usually presents a vector with *N* columns according to the number of scattering angles, while each column contains the respective XRPD signal intensity that was measured. Thus, a given dataset with *p* XRPD files results in an X-matrix of *p × N* variables. To apply PLS, a PLS model that is based on calibration samples has to be constructed. For each diffractogram of the calibration set, the responses (the molar fractions) have to be known. These responses will then form the Y-matrix. PLS now detects the most important components in the data matrix by maximizing the covariance between the X- and Y-matrix [37]. For the construction of a PLS model, it is important to choose the correct number of PLS components to be included in the model. A too small number of PLS components will lead to a poor model performance, while too many PLS components would result in overfitting [39]. To determine the optimal number of PLS components, the method provided by The Unscrambler X software was used, which is based on the minimization of the root mean square error of validation [39,40].

#### 3.2.2. Preparation of the PLS Calibration Set

**Figure 8:**
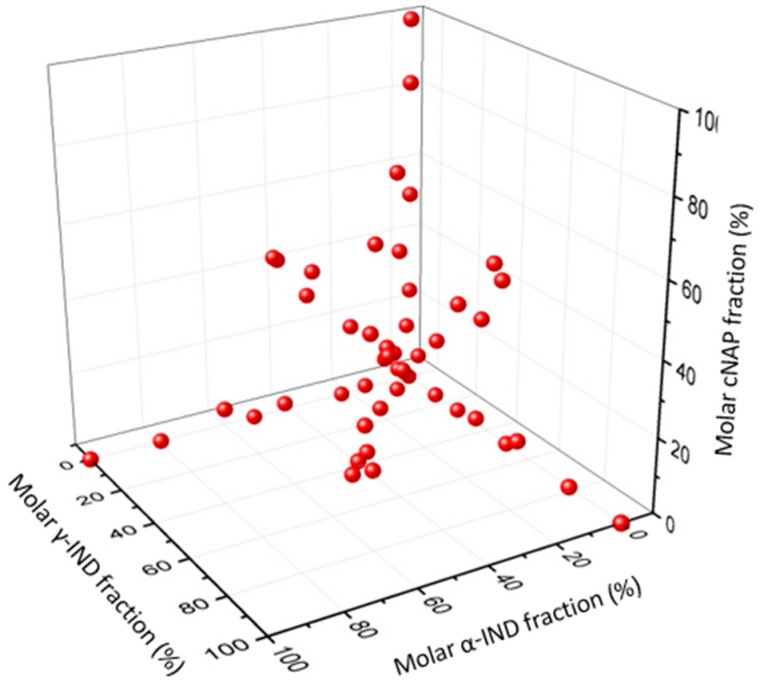
Overview of the phase compositions of the prepared calibration samples containing binary, ternary and quaternary blends of cNAP, γ-IND, α-IND and aNAP/IND_1:1_. The co-amorphous fraction in the samples is determined by the crystalline fractions.

The calibration set has to cover various possible quantitative combinations of the four solid state phases. Binary, ternary and quaternary physical blends comprising cNAP, γ-IND, α-IND and aNAP/IND_1:1_ were prepared. For each of the 52 calibration samples, a total mass of 300 mg with varying molar ratios according to Figure 8 was directly weighed into ball-milling steel jars (see Table A1 in the Appendix for a detailed composition of the calibration samples).

Subsequently, three steel balls with 5 mm diameter were added to the powders before the filled jars were transferred into a freezer at −18 °C for 10 min. This was necessary to ensure that the temperature of the solid was below the glass transition temperature of the co-amorphous portion of the samples, hereby avoiding problems such as suboptimal mixing and incomplete recovery of the powders due to the sticky character of aNAP/IND above its T_g_.

Immediately thereafter, the cooled milling jars were attached to a Retsch MM 200 ball mill (Retsch GmbH, Haan, Germany) and blending was performed at 10 Hz for 1 min until a homogeneous powder was obtained. The powders were transferred into glass tubes and analyzed by XRPD in duplicate. To ensure that no crystalline-to-amorphous or amorphous-to-crystalline transformations of the compounds occur during the blending process, 300 mg of each plain compound was treated in the same manner as the calibration samples. Comparison of the diffractograms of treated and untreated samples revealed no differences (data not shown).

#### 3.2.3. X-ray Powder Diffractometry

XRPD analysis was performed using an X‘Pert PRO X-ray diffractometer (PANalytical, Almelo, The Netherlands; Cu Kα anode; λ = 1.5406 Å; 45 kV; 40 mA). Samples were placed onto an aluminium sample holder and gently compressed with a glass plate to obtain a compact powder with a flat surface. The samples were scanned in duplicate in reflection mode between 5 and 35° 2θ with a scan speed of 0.045° 2θ/min and a step size of 0.0131° 2θ (see the supplementary information for the raw diffractograms).

To prepare the XRPD data for the PLS model construction, all obtained diffractograms were separately baseline offset corrected and normalized to unit area [41] using The Unscrambler X software (ver. 10.3, CAMO Software, Oslo, Norway). Normalization presents an important preprocessing step to increase the PLS model performances, as the intensity of the diffractograms for an identically composed sample can vary due to alterations of the experimental conditions, such as the amount of powder that is placed onto the aluminium holder or the resulting powder density [37]. Subsequently, using Microsoft Excel all duplicate diffractograms were corrected for systematic peak shifts along the °2θ axis before averaging was performed. Hereafter, four PLS regression models, one for each of the solid state phases (cNAP, γ-IND, α-IND and aNAP/IND), were constructed based on the processed diffractograms. For the determination of the area under the Bragg peaks, the background determination function in the Highscore Plus software (ver. 2.2e, PANalytical) was used.

#### 3.2.4. Cross Validation of the PLS Models

To determine the predictive quality of the PLS models, leave-one-out cross validation was performed for the PLS calibration set using The Unscrambler X software. Hereby, the 52-samples PLS calibration set was separated into a 51-samples training set and the test sample. The construction of the PLS models was performed based on the training dataset and subsequently the test sample was predicted based on these PLS models. This procedure was repeated 52 times until each sample was left out once [39]. Finally, all predictions were combined to calculate R^2^ and the RMSE.

## 4. Conclusions

The presented results show that the X-ray powder diffractometry (XRPD) data-based multivariate quantification of up to four simultaneously present solid state phases involving three crystalline and one co-amorphous phase is possible by application of one partial least square (PLS) regression model for each of the four phases. The root mean square errors (RMSE) during the leave-one-out cross validations for the predictions of the crystalline components in the linear areas are found to be between 3.11% and 3.45% and are thus comparable to results reported for the determination of one crystalline phase in binary mixtures with an amorphous phase [32] and for the quantification of the fractions in quaternary mixtures involving exclusively crystalline compounds [37]. Furthermore, PLS prediction of the co-amorphous fraction in the calibration samples was also possible with a slightly increased RMSE of 5.57%. In a future study, based on the present PLS models, the recrystallization behavior of co-amorphous naproxen-indomethacin in dependence of the composition of the co-amorphous phase and the preparation method will be investigated.

## Supplementary Materials

Supplementary materials can be accessed at: http://www.mdpi.com/1420-3049/20/10/19571/s1.

## Appendix

**Table A1:** Molar compositions of the 52 prepared calibration samples.

Sample	F_cNAP_ (%)	F_γIND_ (%)	F_αIND_ (%)	F_am_ (%)
1	3.6	3.6	3.9	88.9
2	4.2	3.1	4.5	88.1
3	8.1	10.7	8.8	72.4
4	7.9	9.1	9.4	73.6
5	17.6	18.6	16.6	47.2
6	15.8	15.7	17.1	51.4
7	22.9	24.9	22.7	29.5
8	21.6	27	23.6	27.8
9	29.5	28.3	29.9	12.2
10	0	37.7	39.4	22.9
11	0	34.4	34.7	30.8
12	0	43.4	44.5	12.1
13	0	44.2	38.8	17
14	30	36.5	0	33.5
15	43.7	45.6	0	10.6
16	47	41.8	0	11.1
17	37.3	0	31.6	31.1
18	31	0	33.4	35.6
19	42.7	0	42.4	14.9
20	43.6	0	43.5	12.8
21	0	0	50.7	49.3
22	0	0	79.3	20.7
23	0	49.6	0	50.4
24	0	78.1	0	21.9
25	53	0	0	47
26	83.3	0	0	16.7
27	62.8	7.9	9.1	20.3
28	8.6	8.3	63.8	19.3
29	8.9	64.9	7.4	18.8
30	8.5	35.9	36.2	19.4
31	35.5	36	7.3	21.2
32	45.3	9.8	17.2	27.6
33	4.6	5.9	44.1	45.4
34	39.6	4.7	6.3	49.5
35	5.3	42	4.9	47.8
36	5	22.8	23.5	48.7
37	20	22.5	5.2	52.3
38	21.7	5.3	22.6	50.4
39	1.8	2.1	16	80.1
40	16.3	1.7	2	80.1
41	1.6	17.2	1.9	79.3
42	1.7	8.6	9.3	80.3
43	10.2	9	2.6	78.2
44	8.1	2	9.5	80.3
45	0	0	22.6	77.4
46	0	25.7	0	74.3
47	25.7	0	0	74.3
48	0	0	100	0
49	0	100	0	0
50	100	0	0	0
51	0	0	0	100
52	0	0	0	100
